# Cervical Cancer Screening: Histologic Outcomes of HPV-Negative HSIL/ASC-H Cytology in a Tertiary Referral Cohort in Northern Thailand

**DOI:** 10.3390/medicina62020371

**Published:** 2026-02-13

**Authors:** Sopita Prasertpakdi, Prapaporn Suprasert, Tanadon Salakphet, Surapan Khunamornpong

**Affiliations:** 1 Division of Gynecologic Oncology, Department of Obstetrics and Gynecology, Faculty of Medicine, Chiang Mai University, Chiang Mai 50200, Thailand; sopita.pra@cmu.ac.th (S.P.); tanadon.s@cmu.ac.th (T.S.); 2 Division of Gynecologic Pathology, Department of Pathology, Faculty of Medicine, Chiangmai University, Chiang Mai 50200, Thailand; skhunamo@yahoo.com

**Keywords:** cervical cancer screening, high-grade squamous intraepithelial lesion, atypical squamous cells, cannot exclude HSIL, human papillomavirus, HPV-negative, colposcopy, cervical intraepithelial neoplasia, p16 Immunohistochemistry

## Abstract

*Background and Objectives*: Cotesting combines cervical cytology and HPV testing and usually identifies HSIL/ASC-H in association with HPV positivity; however, a small subset shows discordant results with high-grade cytology but negative HPV testing. We evaluated the clinicopathologic significance and histologic outcomes of HPV-negative HSIL or ASC-H cytology in a tertiary referral setting. *Materials and Methods*: We retrospectively reviewed women referred to a tertiary colposcopy unit (January 2019–October 2025) with HPV-negative HSIL or ASC-H on cotesting. Clinical findings, colposcopy, histology, excisional procedures, and follow-up were abstracted. Cytology and histology were reviewed by an expert gynecologic pathologist, and p16 immunohistochemistry was performed in all cases. *Results*: Among 92 women with HSIL/ASC-H cytology who underwent cotesting, 84 were HPV-positive (35 HSIL, 49 ASC-H). Eight cases (8.7%) remained HPV-negative after cytology review: 2/37 (5.4%) HSIL and 6/55 (10.9%) ASC-H. On histology, 4/8 (50%) had HSIL (CIN3) and 4/8 had LSIL; all CIN3 cases showed diffuse block-type p16 positivity. Two of six HPV-negative ASC-H cases (33.3%) were CIN3. One patient had persistent high-grade disease requiring two excisional procedures during follow-up. *Conclusions*: HPV-negative HSIL/ASC-H cytology is uncommon but associated with a substantial risk of CIN3. The consistent p16 positivity in tissue-confirmed HSIL supports HPV-attributable disease and suggests that most discordant cases reflect false-negative HPV testing rather than HPV-independent pathology. High-grade cytology should prompt colposcopic evaluation regardless of HPV status, and management should not be de-escalated solely on the basis of a negative HPV test.

## 1. Introduction

Cervical cancer is the fourth most common cancer among women worldwide, with an estimated 660,000 new cases and approximately 350,000 deaths in 2022; nearly 94% of deaths occur in low- and middle-income countries [1]. Persistent infection with high-risk human papillomavirus (HPV) is a necessary cause of almost all cervical cancers, making the disease highly preventable through vaccination and effective screening [2].

Over the past decade, cervical cancer screening has progressively shifted from cytology-based approaches toward HPV-based strategies. Several international guidelines now accept multiple screening options but increasingly favor primary HPV testing with cytology triage for routine screening because of its favorable balance between sensitivity, specificity, and resource utilization [3,4]. National recommendations in Thailand similarly endorse HPV-based screening as a primary strategy [5].

Although cotesting (cytology plus HPV testing) can increase detection of prevalent high-grade lesions, this comes at the cost of reduced specificity and increased downstream testing and colposcopy. Consequently, many screening programs and guideline groups emphasize primary HPV testing with cytology triage rather than routine cotesting in population-based screening, particularly in low-prevalence and vaccinated populations [3,6,7,8].

A clinically important challenge arises when cytology demonstrates high-grade squamous intraepithelial lesion (HSIL) or atypical squamous cells cannot exclude HSIL (ASC-H) in the setting of a negative HPV test. Although such discordant results are uncommon, multiple studies have shown that a meaningful proportion of affected women harbor tissue-confirmed high-grade disease [9,10,11,12,13,14,15,16,17,18,19,20]. In most cases, HPV negativity reflects false-negative testing due to limited cellularity, low viral load, pre-analytical factors, or HPV types not included in the assay rather than truly HPV-independent disease [21,22,23,24,25,26,27,28]. Importantly, HPV-negative results may lead to false reassurance if management is de-escalated, whereas current clinical practice supports colposcopic evaluation for high-grade cytology regardless of HPV status [3,6].

This issue is particularly relevant in low-resource and high–HPV-burden settings, where access to repeated screening, timely follow-up, and colposcopy services may be limited. In such contexts, reliance on a negative HPV result may lead to false reassurance and inadvertently delay referral or diagnostic evaluation, increasing the risk of missed or delayed diagnosis of clinically significant cervical precancer.

To address the limited regional data, we conducted a retrospective study evaluating the clinical characteristics and histopathologic outcomes of women with HPV-negative co-testing but high-grade cytology (HSIL or ASC-H) at a tertiary referral center in Northern Thailand. This study is intended to inform clinical management after identification of high-grade cytologic abnormalities, rather than to support routine co-testing in population-based primary screening.

At the population level, primary HPV screening with cytology triage is generally favored over routine cotesting, as cotesting provides only modest incremental detection while substantially increasing testing and colposcopy burden, particularly in low-prevalence screening populations [7,8,29]. Accordingly, our findings should be interpreted within the context of post-screening diagnostic evaluation rather than screening strategy selection.

## 2. Materials and Methods

### 2.1. Study Design and Ethical Approval

This retrospective study was approved by the Institutional Ethics Committee of Chiang Mai University (OBG-2568-0501). Medical records of all women who were referred to and attended the tertiary colposcopy clinic at Chiang Mai University Hospital between January 2019 and October 2025 were reviewed. Eligible patients were those whose cotesting results demonstrated negative high-risk HPV testing with concurrent HSIL or ASC-H cytology.

For cotesting, cervical cytology and high-risk HPV testing were generally performed on the same liquid-based cytology (LBC) specimen collected during the same clinical visit. In a small number of cases, cervical cytology was obtained using a conventional Pap smear with HPV testing performed separately. In one patient, high-risk HPV testing was conducted approximately four months after the initial ASC-H cytologic result and was reported as negative.

Patients were identified from colposcopy clinic records following referral from multiple institutions within the regional healthcare network or from screening performed at our institute. Patients were excluded if they (1) lacked histologic confirmation or (2) had a history of prior treatment for cervical intraepithelial neoplasia (CIN).

### 2.2. HPV Testing and Cytology Evaluation

HPV testing and cytologic evaluation were performed at the referring institutions before referral to the colposcopy clinic. The types of HPV assays used at the originating laboratories were recorded, and cytologic slides were requested for centralized review when available. All cytologic interpretations were performed in accordance with the Bethesda System and subsequently reviewed by an experienced gynecologic pathologist (SK) to ensure diagnostic consistency.

### 2.3. Histopathologic Assessment and p16 Immunohistochemistry

Biopsy and excisional specimens were evaluated according to the World Health Organization (WHO) classification [30] and the Lower Anogenital Squamous Terminology (LAST) Project criteria [31]. To confirm the histologic diagnosis of HSIL, p16 immunohistochemistry (IHC) was performed in all cases using the CINtec^®^ p16 Histology assay (Roche Diagnostics, Mannheim, Germany) on the VENTANA BenchMark ULTRA automated staining platform with VENTANA detection kits (Roche Diagnostics). A positive p16 result was defined as a diffuse, block-type pattern, characterized by continuous strong nuclear and cytoplasmic staining extending from the basal layer beyond the lower third of epithelial thickness [30,31,32].

### 2.4. Clinical and Colposcopic Data Collection

Demographic data, screening history, colposcopic findings, results of colposcopy-directed biopsies (CDB), loop electrosurgical excision procedures (LEEP), and follow-up outcomes were systematically extracted from electronic medical records. Colposcopic impressions were classified in accordance with the International Federation for Cervical Pathology and Colposcopy (IFCPC) terminology [9]. Patients with a final diagnosis of CIN were followed according to institutional protocols, consisting of cytologic surveillance every 6 months for two consecutive visits, followed by annual cytology if no abnormalities were detected.

### 2.5. Data Management and Statistical Analysis

Data were anonymized and stored in a secure, password-protected research database. Descriptive statistics were used to summarize clinical and histologic outcomes. Due to the small sample size of the discordant cohort, data were summarized using descriptive statistics only, and no inferential statistical tests were applied.

## 3. Results

During the study period, 3342 patients attended the colposcopy unit, reflecting a selected referral population enriched for abnormal screening results. Of these, 242 (7.2%) had HSIL cytology, and 238 (7.1%) had ASC-H cytology. These proportions are higher than expected in population-based screening and reflect pre-referral clinical triage rather than primary screening prevalence. Among patients with high-grade cytology, 92 had available cotesting results. Within this subgroup, 84 patients were HPV-positive, including 35 HSIL and 49 ASC-H cases. The remaining 8 patients were HPV-negative, comprising 3 HSIL cases (7.9% of HSIL) and 5 ASC-H cases (9.3% of ASC-H).

Among the eight HPV-negative cases, seven underwent HPV testing using the Roche cobas assay, while HPV testing in one case (SN2) was performed at a referring institution where the specific assay platform was not documented in the official report. Cotesting was performed on the same liquid-based cytology (LBC) specimen in six cases.

In the remaining two cases (SN4 and SN7), cervical cytology was obtained using a conventional Pap smear. In case SN7, cytology and HPV testing were performed during the same clinical visit. In case SN4, high-risk HPV testing was performed approximately four months after the initial ASC-H cytologic result and was reported as negative. The patient was initially followed conservatively at the referring institution with repeat cytology at three months, which was negative. A subsequent Pap smear at six months demonstrated recurrent ASC-H, prompting referral to our center for colposcopic evaluation, which ultimately revealed low-grade squamous intraepithelial lesion (LSIL). The patient subsequently received HPV vaccination following the second follow-up cytologic assessment.

**Figure 1:**
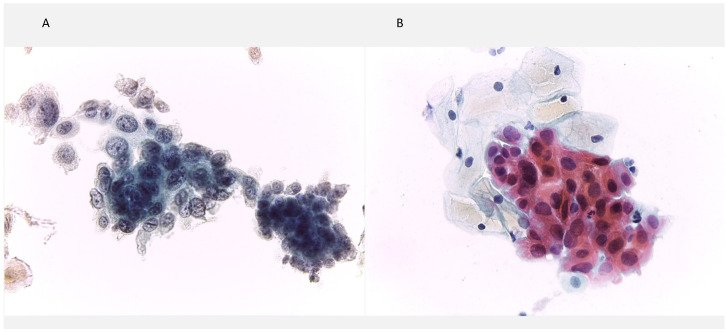
Cervical cytology findings. (**A**) High-grade squamous intraepithelial lesion (HSIL), showing syncytial groups of atypical squamous cells with a high nuclear-to-cytoplasmic ratio and irregular, hyperchromatic nuclei (Papanicolaou stain, ×400). (**B**) Atypical squamous cells, cannot be excluded HSIL (ASC-H), characterized by atypical squamous cells with enlarged, irregular, hyperchromatic nuclei and variable nuclear-to-cytoplasmic ratios, consistent with ASC-H in a background of low-grade squamous intraepithelial lesion (Papanicolaou stain, ×400).

Detailed clinical and pathologic characteristics of all patients are summarized in Table 1, and representative cytologic features of HSIL and ASC-H are shown in Figure 1A and Figure 1B, respectively.

The mean age of the eight patients was 49.6 ± 10.9 years. Colposcopic impressions were recorded as HSIL in 5 cases and LSIL in 3 cases. The median interval between cotesting and colposcopy was 32.5 days (range 18–63 days). Diagnostic procedures included LEEP in 6 patients, CDB alone in 1, and CDB followed by LEEP in 1 patient.

### 3.1. Final Histology Outcomes

Final histologic evaluation identified HSIL (CIN 3) in 4 patients and LSIL in 4 patients, resulting in an overall HSIL prevalence of 50% among women with HPV negative high-grade cytology. All cases with a final diagnosis of HSIL demonstrated strong, diffuse block-type p16 IHC positivity, supporting HPV-related pathogenesis.

One patient (SN7), initially reported as having HSIL cytology with a corresponding HSIL colposcopic impression, underwent LEEP, which revealed low-grade squamous cell intraepithelial lesion (LSIL) with negative p16 immunostaining. Subsequent cytologic review led to revision of the original cytologic interpretation to ASC-H. No diagnostic changes were identified following cytologic review in the remaining cases.

The median follow-up duration for the cohort was 11.8 months (range, 6.3–69.7 months). Notably, the patient with persistent HSIL (SN1) was followed for approximately 60 months, during which repeat excisional treatment was required.

### 3.2. Patients with HSIL Cytology

Two patients presented with HSIL cytology and both had the final diagnosis of HSIL.

**Figure 2:**
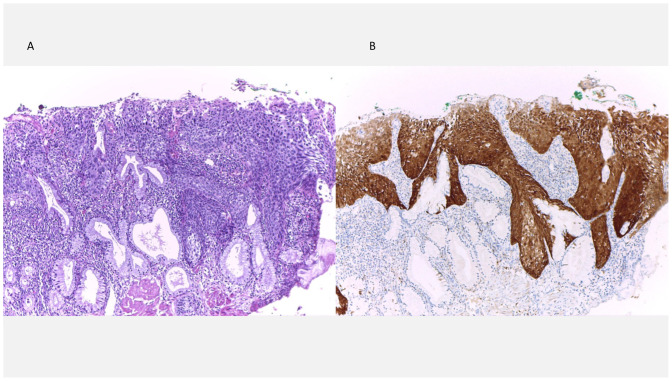
HSIL in LEEP specimen. (**A**) Atypical immature basal-type squamous cells involving the entire epithelial thickness (CIN3), with extension into endocervical glands and a positive endocervical margin (Hematoxylin and eosin stain, ×100). (**B**) p16 immunohistochemistry shows diffuse, strong block-type positivity in the full epithelial thickness (×100).

- Patient SN1, age 41, underwent same-day colposcopy and LEEP, which demonstrated CIN3 involving all quadrants with positive ectocervical and endocervical margins. p16 staining was strongly positive (Figure 2). At the 6-month follow-up, cytology again revealed HSIL, prompting a repeat colposcopy and colposcopic-directed biopsy (CDB), which confirmed the presence of HSIL. She subsequently underwent a second LEEP, which again revealed HSIL with positive surgical margins. She continued surveillance with liquid-based cytology every 6 months, and her most recent cotest in August 2025 was negative.

**Figure 3:**
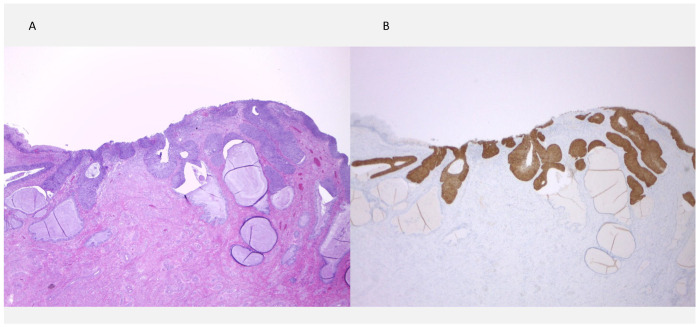
HSIL in LEEP specimen. (**A**) Basal-type atypia involving the entire thickness of the squamous epithelium (CIN3), with endocervical glandular involvement (Hematoxylin and eosin stain, ×25). (**B**) p16 immunohistochemistry showing diffuse, strong block-type positivity (×25).

○ Patient SN6, age 44, had a colposcopic impression of HSIL and underwent LEEP within 3 days. The specimen demonstrated CIN3 with positive margins, and p16 staining was strongly positive (Figure 3). A repeat LEEP performed 6 weeks later revealed LSIL with negative margins. Six-month follow-up cytology was negative.

### 3.3. Patients with ASC-H Cytology

Among the six ASC-H cases, two (33.3%) had a final diagnosis of HSIL:

- Patient SN3, age 43, had an HSIL colposcopic impression and underwent LEEP, revealing CIN3 and CIN2 with negative margins.
- Patient SN5, age 52, had LSIL colposcopic impression; initial CDB demonstrated HSIL, and subsequent LEEP confirmed CIN3 with free margins.

The remaining four ASC-H cases demonstrated LSIL (CIN1) on final histology. Positive p16 IHC was observed in one patient despite a typical LSIL histomorphology.

### 3.4. Summary of Key Diagnosis Findings

- Following cytologic review, HPV negativity persisted in 5.4% (2/37) of HSIL and 10.9% (6/55) of ASC-H cases, indicating that discordant cotesting remains clinically relevant.
- All HPV-negative HSIL cytology cases (100%) were ultimately confirmed as HPV-associated HSIL, supported by p16 positivity.
- Among ASC-H cytology with negative HPV, 33.3% (2/6) had HSIL (CIN3).

## 4. Discussion

In this tertiary colposcopic referral cohort, we evaluated the clinical and histopathologic significance of discordant cotesting results defined as high-grade cytology (HSIL or ASC-H) with a negative high-risk HPV test. Although HPV negativity occurred in only a small proportion of high-grade cytology cases (5.4% of HSIL and 10.9% of ASC-H), the histologic yield of clinically significant disease was substantial. Half of all discordant cases were confirmed as HSIL (CIN3), and all CIN3 diagnoses were supported by diffuse block-type p16 immunohistochemistry, indicating HPV-related oncogenesis despite negative molecular testing. Among HPV-negative ASC-H cases, one-third harbored CIN3, underscoring that HPV negativity does not exclude high-grade disease in this clinical context.

Importantly, this cohort represents a highly selected tertiary colposcopic referral population enriched for abnormal screening results rather than a primary cervical cancer screening population. Therefore, the observed prevalence of HPV-negative HSIL/ASC-H should not be interpreted as reflecting population-based screening performance, predictive values, or test sensitivity. Nonetheless, the finding of persistent high-grade disease in one HPV-negative patient requiring two excisional procedures over nearly five years highlights that HPV negativity should not be interpreted as reassurance when cytology suggests HSIL or ASC-H. Clinically, these findings reinforce current management principles: high-grade cytology warrants prompt colposcopic evaluation regardless of HPV status, and management should not be de-escalated solely because the HPV test is negative.

Our findings are consistent with prior reports demonstrating that HPV-negative HSIL/ASC-H is uncommon but clinically meaningful, with substantial heterogeneity in reported discordance rates across studies. To contextualize our results, we compared our findings with previously published studies examining discordant high-grade cytology with negative HPV testing, summarized in Table 2. Across studies reporting cytology-based discordance, the prevalence of HPV-negative HSIL cytology ranged from 3.7% to 20.2%, while HPV-negative ASC-H cytology ranged from 2.5% to 44.2% [9,10,11,12,13,14,15,16,17,18,19,20,33]. Studies evaluating broader categories of abnormal cytology reported discordance rates between 2.3% and 14.5% [10,33]. This wide variability likely reflects differences in study populations (screening versus referral settings), cytologic thresholds, referral pathways, and laboratory practices. In particular, the highly selected nature of our referral cohort limits direct generalizability to population-based screening settings.

In addition, multiple studies focusing on tissue-confirmed outcomes have shown that approximately 4.5–8.3% of histologically confirmed HSILs were HPV-negative on initial testing [21,22,23,24,25,26,27], closely aligning with our tissue-based findings. Several investigations further demonstrated that many initially HPV-negative HSILs were subsequently confirmed as HPV-related through tissue-based molecular assays or p16 immunohistochemistry [11,21,22,23,24,25,26], highlighting the importance of adjunctive diagnostics in resolving apparent discordance.

The observed heterogeneity across studies is partly explained by differences in HPV testing platforms, including Hybrid Capture 2, Roche cobas, and Aptima assays, each with distinct analytical sensitivity, molecular targets, and genotype coverage [10,11,12,13,14,15,16,17,18,19,20,21,22,23,24,25,26,27,33]. In our cohort, nearly all patients underwent HPV testing using the Roche cobas assay, which targets 14 high-risk HPV genotypes. Assay-related limitations—such as relatively high clinical cut-off thresholds designed to avoid detection of transient or very low viral loads, suboptimal sampling, DNA degradation, or technical inhibition—represent major contributors to false-negative HPV results. Certain HPV genotypes may also escape detection because they are not included in specific assay panels or due to sequence variation within targeted genomic regions. However, although additional HPV types beyond the standard high-risk panel have been detected in cervical cancers, Arbyn et al. demonstrated that these types are rare and contribute only minimally to the overall cervical cancer burden, concluding that expansion of screening panels beyond currently validated high-risk HPV types is not warranted [34]. Accordingly, incomplete genotype coverage alone is unlikely to fully explain HPV-negative high-grade lesions observed in clinical practice.

From a mechanistic perspective, Prétet et al. comprehensively summarized the causes of HPV-negative high-grade lesions into sample-related, clinicopathologic, assay-related, and biologic factors [28]. Sample-related issues include limited lesional volume, focal HSIL involvement, or insufficient infected cells, all of which may lead to false-negative molecular results despite biologically significant disease. Clinicopathologic factors, such as misclassification of lesion origin or reactive and atrophic changes mimicking high-grade lesions, may also contribute, emphasizing the value of centralized expert pathology review and systematic p16 immunohistochemistry, as performed in our study.

Although rare, true HPV-negative high-grade lesions have been described, including clearance of HPV DNA before lesion regression (“hit-and-run” hypothesis) and truly HPV-independent oncogenic pathways [28]. However, in our cohort, the uniform block-type p16 positivity in all tissue-confirmed HSIL cases strongly supports HPV-driven oncogenesis, suggesting that most discordant cases are more consistent with false-negative HPV testing rather than truly HPV-independent disease.

Long-term progression risk is strongly HPV genotype–dependent. Large longitudinal studies have consistently demonstrated that lesions associated with HPV16, and to a lesser extent HPV18, carry substantially higher risks of CIN3+ and cervical cancer compared with other HPV types [35,36]. Accordingly, HPV-negative CIN2/CIN3 lesions—often reflecting non-16/18 genotypes or very low viral load—may on average carry a lower subsequent cancer risk than HPV16-associated disease. However, this distinction does not support the de-escalation of management when high-grade cytology is present. Post hoc analyses from the ATHENA trial demonstrated that most cobas-negative CIN2+ lesions remained HPV-attributable upon expert slide review and ancillary testing, including p16 immunohistochemistry and tissue-based PCR, and no convincing cases of truly HPV-independent CIN3 or adenocarcinoma in situ were identified [23].

The implications of our findings should therefore be interpreted within the appropriate clinical context. Our data primarily inform post-screening diagnostic evaluation rather than the selection of population-based screening strategies. We do not intend to imply support for routine cotesting in population-based primary screening. Indeed, contemporary guidelines favor primary HPV testing with cytology triage, reflecting evidence that routine cotesting provides only modest incremental detection while substantially increasing colposcopy burden and downstream procedures [7,8,29]. Population-based evidence from the Kaiser Permanente Northern California cohort further demonstrated that although cotesting detects additional CIN2+ lesions, clinically meaningful false-negative results still occur in both components, and HPV-negative high-grade cytology remains a significant clinical scenario that should not prompt de-escalation of management [6].

CIN2 represents a heterogeneous histologic endpoint with variable potential for regression or progression. In the ASCUS–LSIL Triage Study, approximately 40% of CIN2 lesions regressed spontaneously over two years, with regression occurring less frequently in lesions associated with HPV16 [37]. While this heterogeneity raises concerns about overtreatment, particularly for CIN2, the presence of high-grade cytology (HSIL or ASC-H) in a tertiary referral setting warrants careful diagnostic evaluation because of the substantial risk of underlying CIN3.

Genotype coverage and population-level vaccination status further influence the balance of benefits and harms in HPV-based screening. Evidence from Nordic and post-vaccination cohorts suggests that restricting screening to genotypes included in the nonavalent vaccine may improve specificity with minimal loss in cancer detection, as HPV16/18/31/33/45/52 account for the majority of screen-detected invasive cervical cancers, whereas additional genotypes included in broader assays contribute only marginally to cancer prevention [38,39,40]. In HPV-vaccinated populations, broad 14-genotype testing and routine co-testing may therefore yield diminishing returns and increase downstream follow-up for a small incremental gain in cancer detection. Nevertheless, even in vaccinated settings, HPV-negative high-grade cytology remains clinically significant in referral populations and should prompt standard colposcopic evaluation.

In regions where HPV-based screening is being implemented, particularly in tertiary-care and referral settings such as Northern Thailand, our findings have important clinical and guideline implications. While primary HPV testing with cytology triage remains the preferred population-based strategy, discordant HPV-negative high-grade cytology represents a scenario in which reliance on HPV status alone may lead to false reassurance. In settings with limited access to repeated screening or delayed referral pathways, de-escalation of management based solely on HPV negativity may increase the risk of missed or delayed diagnosis of high-grade cervical precancer. Our results, therefore, support regional guideline adaptation that emphasizes cytologic severity and histologic confirmation in post-screening clinical decision-making, particularly within tertiary referral centers.

This study has several strengths. It addresses a clinically relevant yet relatively uncommon discordant scenario using detailed clinicopathologic correlation in a real-world tertiary referral setting. Centralized expert review of cytology and histology, together with systematic application of p16 immunohistochemistry, strengthens diagnostic accuracy. In addition, longitudinal follow-up, including repeat excisional procedures in selected cases, demonstrates that clinically significant and persistent high-grade disease can occur despite HPV negativity.

Several limitations should be acknowledged. This was a retrospective, single-center study conducted in a tertiary referral setting with a small number of discordant cases, limiting statistical power and generalizability. Screening denominators and referral pathways were decentralized across multiple institutions, precluding population-level estimates of test performance. HPV testing platforms were not entirely uniform, although nearly all cases were tested using the Roche cobas assay. In addition, while cotesting was performed on the same liquid-based cytology specimen in most patients, two cases underwent conventional Pap smear cytology with HPV testing performed separately at referring institutions; this minor methodological variation is unlikely to have materially influenced the overall findings. Tissue-based HPV genotyping was not routinely available, and HPV vaccination status before screening or after treatment was not consistently documented and was unavailable for most patients, precluding evaluation of the potential impact of vaccination on discordant cotesting results. Finally, long-term oncologic outcomes such as progression to invasive cervical cancer were not assessed. Larger, prospective, multicenter studies with standardized testing and extended follow-up are needed to refine absolute risk estimates and validate these findings.

## 5. Conclusions

In conclusion, HSIL or ASC-H cytology with a negative HPV test is uncommon but clinically meaningful. In this tertiary referral cohort, all HPV-negative HSIL cytology cases and one-third of HPV-negative ASC-H cases were confirmed as HSIL (CIN3) on histology, supported by diffuse block-type p16 positivity, indicating that most discordant results reflect false-negative HPV testing (and/or HPV types not covered by the assay) rather than truly HPV-independent disease. Clinically significant and even persistent high-grade lesions may occur despite HPV negativity, supporting prompt colposcopic evaluation with biopsy and management guided by cytologic severity and tissue diagnosis rather than HPV status alone. These findings reinforce current guideline principles that high-grade cytology warrants definitive diagnostic evaluation regardless of HPV test results and caution against de-escalation of management based solely on a negative HPV test.

## Figures and Tables

**Table 1:** Summary of cytology, colposcopy, and Histology outcomes in case HPV negative.

SN	Age (Years)	Type of HPV Test	Cytology:Type (Date)	Colposcopy Impression (Date)	Interval Time (Days)	Procedure (Date)	CDB Result	Final Histology	p16	Risk Factors	FU Duration (Months)	Note
1 DB	41	Cobas	HSIL:LBC (28/10/19)	HSIL (26/11/19)	29	LEEP (26/11/19)	-	HSIL (CIN3, all quadrant, endocervical margin positive, ectocervical margin positive)	positive	2 partners 1st SI age 22 years	69.7	FU 6 mos -> LBC: HSIL -> colpo -> CDB (2/6/20)HSIL -> 2nd LEEP (3/7/20) HSIL positive endo&ecto cervical margin -> LBC 25/8/20 neg, CDB neg -> FU q 6 mos all LBC neg -> DLV cotest 19/8/25 HPV negative, cytology negative
2 JP	35	Unknown	ASC-H:LBC (14/5/20)	LSIL (23/6/20)	40	CDB (25/6/20)	LSIL	LSIL	positive	1 partner 1st SI age 20 years	11.8	FU DLV 15/6/21 LBC: negative
3 NJ	43	Cobas	ASC-H:LBC (27/1/23)	HSIL (28/2/23)	32	LEEP (17/3/23)	-	HSIL free margin (CIN2,3)	positive	4 partners 1st SI age 23 years	NA	-
4 PC	68	Cobas	ASC-H:PAP (4/3/25)	HSIL (6/5/25)	63	LEEP (7/5/25)	-	Atypical metaplastic squamous epithelium, consistent with LSIL	negative	1 partner 1st SI age 24 years	6.3	At former hospital: PAP 12/2/24:ASC-H -> HPV test 5/6/24 negative -> PAP 5/9/24:neg -> 4/3/25 ASC-H -> 3 shots of HPV vaccine, last shot 2/9/25 1st LEEP diagnosis: HSIL free margin -> review R/O LSIL -> FU DLV 11/11/25 LBC negative
5 PT	52	Cobas	ASC-H:LBC (15/3/25)	LSIL (17/4/25)	33	CDB (17/4/25) LEEP (25/4/25)	HSIL	HSIL(CIN3) free margin	positive	-	NA	-
6 PW	44	Cobas	HSIL:LBC (10/1/25)	HSIL (28/1/25)	18	LEEP (31/1/25)	-	HSIL(CIN3) all quadrant, endocervical margin positive 10–12 o’clock, releep 2/4/25 atypical metaplastic squamous epithelium, LSIL, margin free	positive	3 partners	8.6	DLV 16/10/25 negative
7 NP	54	Cobas	HSIL:PAP (6/3/24) Review: ASC-H with LSIL	HSIL (30/4/24)	55	LEEP (2/5/24)	-	LSIL(CIN1) free margin	negative	3 partners 1st SI 20 years	18.4	FU 10/10/25 LBC ASC-H -> colpo 4/11/25: LSIL -> CDB -> negative -> plan FU
8 PJ	60	Cobas	ASC-H:LBC (17/9/25)	LSIL (7/10/25)	20	LEEP (8/10/25)	-	LSIL(CIN1) free margin	negative	2 partners	NA	-

HSIL, high-risk human papillomavirus; LEEP, loop electrosurgical excision procedures; CIN, cervical intraepithelial neoplasia; SI, sexual intercourse; mos, months; LBC, liquid based biopsy; PAP, Papanicoaou smear; CDB, colposcopic directed biopsy; colpo, colposcopy; 1st, first; 2nd, second; FU, follow up; neg, negative; DLV, date last visit; ASC-H, atypical squamous cells cannot exclude high-grade squamous intraepithelial lesion; LSIL, low-grade squamous cell intraepithelial lesion; q, every; R/O, rule out; NA, not available; ->, followed by.

**Table 2:** Published Studies reporting discordant High-Grade Cervical Cytology (HSIL/ASC-H) with Negative High-Risk HPV Testing: Cytologic, Histologic, and Tissue-Based Evidence.

No.	Authors (Year)	Prevalence of HPV-Negative High-Grade Lesions (HSIL/ASC-H) Based on Cytology and/or Tissue Diagnosis	HPV Testing Method	Country	p16	Notes
1	Jastania et al. (2006) [21]	Tissue-confirmed HSIL^+^, HPV-negative: 4.5% (10/220)	Hybrid capture 2	Canada	NA	HPV-negative tissue-confirmed HSIL was identified in 4.5% (10/220) of cases.
2	Negri et al. (2007) [10]	Abnormal cytology 2.28% (94/4130)	Hybrid capture 2	Italy	NA	Repeat HPV testing detected high-risk HPV in 46.8% of specimens with initially negative results. Among cases with persistently negative hrHPV, one CIN2 and one invasive carcinoma were identified on histology.
3	Cohen et al. (2012) [11]	Cytology ASC-H 33.4% (1054/3155)	Hybrid capture 2	USA	NA	HSIL was identified in 2.36% (13/549) of HPV-negative ASC-H cases with histologic follow-up.
4	Blatt et al. (2015) [33]	Abnormal cytology 14.5% (37,243/256,648)	Hybrid capture 2	USA	NA	Among abnormal cytology cases with negative HPV testing, 15.3% (571/37,243) were diagnosed with CIN2+, including CIN2 (390 cases), CIN 3 (120 cases, AIS (6 cases), SCCA (26 cases), AdenoCA (29 cases)
5	Chen et a (2015) [12]	Cytology ASC-H 31.8% (157/493)	Hybrid capture 2	USA	NA	Histologic CIN2+ was identified in 8.9% (14/157) of HPV-negative ASC-H cytology cases.
6	Zhang et al. (2015) [22]	Tissue confirmed HSIL 7% (46/657)	Hybrid capture 2	USA	Done	All HPV-negative tissue-diagnosed HSIL cases were confirmed as CIN2 (29 cases) or CIN3 (17 cases), with p16 positivity supporting HPV-related pathogenesis.
7	Hui et al. (2016) [27]	NA	Cobas	USA	NA	Among 4 HPV-negative HSIL cytology cases, 3 showed LSIL with negative tissue HPV testing, while 1 revealed HSIL with HPV18 detected on tissue analysis.
8	Petry et al. (2016) [23]	Tissue-based evaluation: • CIN2 (*n* = 32) Cobas-negative → Amplicor/LA-negative in 12/32, of which 6/12 were p16-positive • CIN3 (*n* = 23) Cobas-negative → Amplicor/LA-negative in 11/23, of which 8/11 were p16-positive	Cobas Amplicor and/or LA		Done	ATHENA trial Cobas-negative CIN2 (*n* = 32) and CIN3 (*n* = 23) cases underwent sequential testing with Amplicor/LA; among Amplicor/LA-negative cases, p16 positivity was observed in 6/12 CIN2 and 8/11 CIN3, supporting HPV-related disease despite negative molecular testing.
9	Fuller et al. (2018) [13]	Thin prep (cytology) ASC-H 30.5% (22/72) >HSIL 9.3% (4/43) SurePath (cytology) ASC-H 39.06% (25/64) >HSIL 20.25% (16/79)	Cobas	USA	NA	Tissue-diagnosed ≥ HSIL: ThinPrep 6.3% (8/127) vs. SurePath 12.1% (16/132); *p* = 0.10.
10	Tracht et al. (2017) [14]	Cytology HSIL 4.6% (3/65)	Cobas	United Kingdom	Done	All HPV-negative HSIL cytology cases were confirmed as HSIL on histology, with diffuse p16 positivity.
11	Ge et al. (2019) [24]	Tissue confirmed HSIL 8.3% (21/252)	Cobas	USA	NA	Among 252 biopsy-confirmed HSIL cases, 8.3% (21/252) were HPV-negative on initial testing; all but one were subsequently confirmed as high-risk HPV–positive by tissue-based molecular analysis.
12	Ashman et al. (2020) [15]	Cytology HSIL Thin prep Overall 6.36% (60/943) Hybrid Capture 2 5.6% (12/214) Cervista 9.2% (22/238) Aptima 5.3% (26/491)	Hybrid Capture 2 (241 cases) Cervista (214 cases) Aptima (491 cases)	USA		Tissue HSIL confirmation: Overall 53.3% (24/45); Hybrid Capture 2 50% (4/8), Cervista 60% (12/20), Aptima 46.0% (8/17), assay-dependent variability observed.
13	Reich et al. (2020) [25]	Tissue confirmed HSIL (374 cases)/AIS (14 cases) 5.1% (20/388)	Aptima Cobas	Austria	Done	All HPV-negative HSIL (19 cases) and AIS (1 case) cases demonstrated p16 positivity, supporting HPV-driven carcinogenesis.
14	Bogani et al. (2021) [16]	Cytology HSIL/ASC-H 14.02% (175/1248)	Hybrid capture 2 Cobas CLART	Italy	NA	Tissue-confirmed HSIL in hrHPV-negative women: 14.9% (260/1738); lower recurrence risk (HR 1.69, 95% CI 1.05–4.80; *p* = 0.018).
15	Borgfeldt et al. (2022) [17]	Cytology ASC-H 2.5% (N = 1/40)	Aptima mRNA	Sweden	NA	HPV-negative ASC-H was rare (1/40) and associated with benign biopsy findings.
16	Li et al. (2021) [18]	Cytology HSIL 3.7% (36/951)	Aptima mRNA	USA	NA	Among 36 HPV-negative HSIL cytology cases, 15 (41.7%) were diagnosed with CIN2+ on histologic evaluation.
17	Agoff et al. (2023) [9]	Cytology HSIL (7)/ASC-H(1)/AGC neoplastic (1) 10.7% (9/84)	Cobas	USA	Done	All cases with HPV-negative high-grade cytology were confirmed on histology as HSIL or invasive disease, including CIN2 (*n* = 2), CIN3 (*n* = 5), AIS with CIN3 (*n* = 1), and squamous cell carcinoma (*n* = 1), with uniform p16 positivity.
18	Karaaslan et al. (2023) [19]	Cytology CA 31.8%(7/22) HSIL 9.2% (73/795) ASC-H 32.8% (291/1074)	Hybrid capture 2	USA	NA	Tissue confirmed Cytology SCCA 2 cases -> CIN3 1 case, SCCA 1 case (abnormal 100%) Cytology HSIL 58 cases -> CA 1 case, CIN 3 13 cases, CIN 2 9 cases (abnormal 39.6%) Cytology ASC-H 189 cases -> CIN 2 9 cases, CIN 3 9 cases (9.5%)
19	Sandeford et al. (2024) [26]	Tissue confirmed HSIL 6.06% (89/1468) (Hybrid capture 2), 5.65% (83/1468) (HPV-ISH)	Hybrid capture 2 HPV-ISH (type 16, 18)	Australia	Done	HPV-negative tissue-confirmed HSIL ranged from 5.7 to 6.1%, with 62.8% p16 positivity
20	Feng et al. (2024) [20]	Cytology Hospital1&2 ASC-H 25.71% (118/459) HSIL 7.17% (17/237) Hospital 3 ASC-H 44.16% (140/317) HSIL 9.93% (14/141)	Aptima	USA	NA	High rates of HPV-negative HSIL and ASC-H cytology were observed across multiple institutions, highlighting persistent diagnostic discordance; however, no data on tissue-confirmed HSIL were reported for HPV-negative cytology cases.

HSIL, high-grade squamous intraepithelial lesion; ASC-H, atypical squamous cells cannot exclude high-grade squamous intraepithelial lesion; HPV, human papillomavirus; NA, not available; hrHPV, high-risk human papillomavirus; USA, United State of America; CIN, cervical intraepithelial neoplasia; 2+, two plus; AIS, adenocarcinoma in situ; SCCA, squamous cell carcinoma; adeoCA, adenocarcinoma; LSIL, low-grade squamous cell intraepithelial lesion; LA, Linear Array HPV Genotyping Test; vs., versus; HR, hazard ratio; CI, confidence interval; AGC, atypical glandular cytology; HPV-ISH, HPV in situ hybridization.

## Data Availability

The data presented in this study are available on request from the corresponding author.

## Abbreviations

The following abbreviations are used in this manuscript:

HPV	Human papillomavirus
hrHPV	High-risk human papillomavirus
HSIL	High-grade squamous intraepithelial lesion
ASC-H	Atypical squamous cells cannot exclude high-grade squamous intraepithelial lesion
ACS	American Cancer Society
ASCCP	American Society for Colposcopy and Cervical Pathology
USPSTF	United States Preventive Services Task Force
CIN3+	Cervical intraepithelial neoplasia grade 3 or more severe results
CIN	Cervical intraepithelial neoplasia
WHO	World Health Organization
LAST	Lower Anogenital Squamous Terminology
IHC	Immunohistochemistry
CDB	Colposcopy-directed biopsies
LEEP	Loop electrosurgical excision procedures
IFCPC	International Federation for Cervical Pathology and Colposcopy
SD	Standard deviation
LSIL	Low-grade squamous cell intraepithelial lesion
